# Changes in the consumption of isoflavones, omega-6, and omega-3 fatty acids in women with metastatic breast cancer adopting a whole-food, plant-based diet: post-hoc analysis of nutrient intake data from an 8-week randomized controlled trial

**DOI:** 10.3389/fnut.2024.1338392

**Published:** 2024-03-21

**Authors:** Jean Lee, Erin K. Campbell, Eva Culakova, Lisa M. Blanchard, Nellie Wixom, Luke J. Peppone, Thomas M. Campbell

**Affiliations:** ^1^Department of Public Health Sciences, University of Rochester Medical Center, Rochester, NY, United States; ^2^Department of Surgery, Cancer Control, University of Rochester Medical Center, Rochester, NY, United States; ^3^Department of Family Medicine, University of Rochester Medical Center, Rochester, NY, United States; ^4^Clinical Research Center, University of Rochester Medical Center, Rochester, NY, United States

**Keywords:** breast cancer, isoflavones, omega-3 polyunsaturated fatty acids, omega-6 polyunsaturated fatty acids, omega-6/-3 ratio, plant-based diet, soy, vegan diet

## Abstract

**Background:**

Diets rich in minimally processed plant-based foods are recommended to breast cancer patients, and some may have an interest in whole-food, plant-based (WFPB) diets that avoid animal-based foods, added fats, and refined sugars. Within WFPB diets, the intakes of isoflavones, omega-6 polyunsaturated fatty acids (n-6 PUFAs), and omega-3 polyunsaturated FAs (n-3 PUFAs), which have been discussed in reference to breast cancer outcomes, have not been well characterized.

**Methods:**

Women with stage IV breast cancer on stable therapy were randomized 2:1 into (1) a WFPB intervention (*N* = 21) or (2) usual care (*N* = 11) for 8 weeks. Three meals per day were provided. Outcomes presented here include dietary intake of isoflavones, n-3 and n-6- PUFAs, which were assessed using three-day food records at baseline and 8 weeks. Baseline and 8-week mean intake within groups were compared using the Wilcoxon signed-rank test and between control and intervention groups by a two-sample *t*-test.

**Results:**

The WFPB intervention participants increased their daily consumption of total isoflavones from a mean of 0.8 mg/day to 14.5 mg/day (*p* < 0.0001) and decreased the n-6:n-3 ratio of their diet from a mean of 9.3 to 3.7 (*p* < 0.0001). Within the WFPB group, linoleic acid (n-6 PUFA) consumption decreased by a mean of 3.8 g (*p* = 0.0095), from 12.8 g/day to 9.0 g/day; total n-3 PUFA consumption increased by a mean of 1.1 g (*p* = 0.0005), from 1.6 g/day to 2.7 g/day.

**Conclusion:**

Transitioning to a WFPB diet resulted in significantly increased isoflavone intake and decreased n-6:n-3 ratio in women with breast cancer.

## 1 Introduction

Patients with breast cancer are often interested in nutrition (1–3) and are commonly recommended to adhere to dietary patterns rich in, but not limited to, minimally processed plant-based foods (4, 5). Plant-rich dietary patterns, or plant-based diets, include a spectrum of different diets, including a Mediterranean diet, Dietary Approaches to Stop Hypertension (DASH) diet, vegetarian, vegan, or a whole-food, plant-based (WFPB) diet. A WFPB diet, studied here, focuses on eating fruits, vegetables, whole grains, legumes, nuts, and seeds while minimizing or fully avoiding animal-based products, added oils, and processed foods.

Beyond dietary patterns, there remain important questions regarding the effect of specific dietary compounds on cancer risks. In particular, patients may ask about isoflavones or certain dietary fats. Isoflavones are found most abundantly in soybeans and foods derived from soybeans. They are a major class of phytoestrogens, which are naturally occurring plant compounds that are structurally similar to estrogens (6). They have a wide range of biologic effects. Phytoestrogens are weakly estrogenic as well as anti-estrogenic and can affect the cell cycle, apoptosis, genetic expression, endogenous steroids, angiogenesis, and also have antioxidant properties (6).

Concern about the potential carcinogenicity of isoflavones was raised based in part, on findings from experimental animal research testing various levels of isolated isoflavones (7–11) and *in vitro* data, but these concerns have not been supported by epidemiologic studies of isoflavones consumed as foods in the diet. A 2006 meta-analysis of 18 cohort and case–control studies found that high soy intake was modestly associated with a reduced risk of breast cancer (12). Three more recent meta-analyses (13–15) also found that high soy isoflavone intake was associated with reduced breast cancer risk in women without breast cancer, though this inverse association was not as strong in cohorts of Western women, who have lower intakes of isoflavones than Asian populations (15). Furthermore, other meta-analyses found that high soy isoflavone intake was associated with better outcomes in women with breast cancer (16, 17).

Similarly, omega-6 polyunsaturated fatty acids (n-6 PUFAs) and omega-3 polyunsaturated fatty acids (n-3 PUFAs), both essential fatty acids, have been studied in cancer risk. N-6 PUFAs are found in widely used vegetable oils like corn and soybean oil as well as nuts and seeds, whereas n-3 PUFAs are found in flax and chia seeds, cold-water fatty fish or fish oils, select plant oils, walnuts and fortified foods (18, 19). It has been suggested that humans evolved on a diet containing an omega-6 to omega-3 PUFAs ratio (n-6:n-3) of 1:1 (20, 21). The modern Western diet, however, has a n-6:n-3 ratio of 10–20:1 (22, 23). One recent meta-analysis shows that a higher n-6:n3 ratio is associated with an increased risk of breast cancer (24), and some research has shown that this fatty acid ratio is associated with inflammation and associated metabolic health (22, 25), but the evidence is far from settled.

Despite the interest in isoflavones and essential fatty acids there has been limited research to characterize the content of these compounds in either the typical Western diet of patients with advanced breast cancer or a WFPB diet. Given the questions around these food compounds, as well as the interest in plant-based diets, the purpose of this paper is to describe the changes in the intake of isoflavones, linoleic acid (n-6 PUFA), and n-3 PUFAs during a randomized controlled trial in women with metastatic breast cancer utilizing a WFPB dietary intervention.

## 2 Materials and methods

We conducted a randomized, controlled trial of a WFPB diet among women with stable stage IV breast cancer undergoing treatment. Our findings for feasibility, quality of life, and cardiometabolic and cancer-related biomarkers are published separately (26, 27). In this sub-analysis, we analyzed isoflavones, linoleic acid (n-6 PUFA), and N-3 PUFAs of the WFPB intervention diet and its n-6:n-3 PUFAs ratio using three-day food records at baseline and 8 weeks (final). Linoleic acid was used as a proxy for measuring total n-6 PUFAs given the vast majority was provided by linoleic acid.

### 2.1 Study selection, inclusion, and exclusion criteria

Study participants were recruited from oncology clinics at the University of Rochester Medical Center and local support groups. Eligibility criteria included: women with stage IV breast cancer with any estrogen receptor, progesterone receptor, and human epidermal growth factor receptor 2 status, who were expected to live at least 6 months by their oncologist, and on a stable treatment regimen for the past 6 weeks with no expected changes in the next 4 weeks, were eligible for the study. Exclusions included inability to tolerate a normal diet, an active malabsorption syndrome, eating disorder, uncontrolled diarrhea, recent consumption of a vegan diet, major surgery within 2 months, current insulin, sulfonylurea, or warfarin use, glomerular filtration rate < 30 mL/min/1.73 m2 or serum potassium >5.3 mmol/L on twice within 90 days, current smoking, illicit drug use, drinking ≥7 alcoholic beverages per week, food allergies or intolerances to plant-based foods, or psychiatric disorder impairing ability to give consent.

### 2.2 Study design

Participants were randomized 2:1 to two arms: a WFPB diet or a usual diet. Given this was a dietary intervention, participants and study staff could not be blinded to the participants’ group assignments. Participants in the control arm were instructed to continue their usual diets for 8 weeks and received phone calls from a study physician at weeks 2 and 6 to assess for adverse events and treatment changes. Participants in the WFPB arm received 3 meals and one side dish per day for 8 weeks and had weekly education visits and a brief weekly phone call with the study physicians. The *ad libitum* WFPB diet consisted of fruits, vegetables, whole grains, legumes, nuts, and seeds. Soy foods and minimal amounts of added sugars were allowed. Every four weeks, there were a total of 84 provided meals (3 meals/day), out of which 16 meals contained small amounts of tofu or soybeans in the entrée (edamame or tofu in a stir fry, or tofu mixed into a sauce, for example). Participants were not required to eat the provided meals and could add their own food in place of or in addition to the provided meals as long as it was ‘on-plan’. The diet excluded animal products and added oils and solid fats. A daily multivitamin (Centrum Women) was provided to all participants in both groups.

### 2.3 Data collection and statistical analysis

Participants completed 3-day food records at baseline and 8 weeks on provided paper forms after instruction from the team’s study coordinator. Participants then received a call from a dietitian within 1–2 days of completing their food record to clarify pertinent details. One intervention participant did not complete a final 3-day food diary and was not included in our analysis. The nutrient contents of 3-day food records were analyzed using the Nutrition Data System for Research (NDSR) version 2017 (Nutrition Coordinating Center, University of Minnesota, Minneapolis, MN) and statistical analysis was performed using SAS 9.4 (SAS Inc., Cary, NC, United States).

Within-group changes in nutrient content between the baseline and final 3-day food records were analyzed using the Wilcoxon signed-rank test, given non-normal distribution. The between-group difference in change of each nutrient was assessed using the Wilcoxon two-sample test. These tests were performed for isoflavones (daidzein, genistein, glycitein, and total isoflavones) and omega PUFAs (linolenic acid, total omega-3 PUFAs, linoleic acid, and linoleic acid: total n-3 PUFAs ratio), kilocalories, and macronutrients. All statistical tests of significance (α < 0.05) were two-tailed.

## 3 Results

Thirty-two participants were enrolled (*n* = 21 WFPB and *n* = 11 control) and 30 completed the study (*n* = 20 WFPB and *n* = 10 control). Table 1 summarizes the energy and macronutrients consumed at baseline and 8 weeks. Within the WFPB group, the intervention resulted in significant changes in energy and the percentage of total kilocalories from fat and carbohydrates (energy and both macronutrients, all *p* < 0.0001) but not protein. Compared to controls, intervention participants had larger changes in both energy (−310.4 ± 111.3 (SE) kcals, *p* = 0.0108) and percentage of total kilocalories from fat (−15.2 ± 3.7% of total kcal, *p* = 0.0044). The control group did not significantly change their diet, except for decreased energy intake (*p* = 0.0371).

**Table 1:** Macronutrients.

	Control diet (CON) *n* = 10			Intervention diet (WFPB) *n* = 19			Between group differences in change at week 8	
	Baseline	8 weeks	Difference	Baseline	8 weeks	Difference	Difference	*p*-value
**Energy (kcal)**								
Mean ± SE	1590.2 ± 140.0	1431.2 ± 126.8	−159.1 ± 63.8*	1782.2 ± 75.8	1312.8 ± 57.4	469.5 ± 73.1***	−310.4 ± 111.3	0.0108
Median (25–75%)	1572.5 (1379.0–2047.5)	1459.4 (995.8–1683.2)	−129.6 (−244.6–1.3)	1752.1 (1468.0–2124.8)	1293.4 (1127.2–1510.2)	−416.0 (−690.6– −220.2)		
**Fat % of total kcal**								
Mean ± SE	36.2 ± 1.8	36.0 ± 4.2	−0.2 ± 3.6	36.9 ± 1.4	21.4 ± 1.2	−15.5 ± 1.9***	−15.3 ± 3.7	0.0044
Median (25–75%)	37.5 (31.8–38.7)	38.6 (29.9–45.2)	1.7 (−4.5–6.1)	36.9 (30.9–42.1)	21.4 (18.3–25.2)	−14.7 (−23.7– −9.9)		
**Carbohydrate % of total kcal**								
Mean ± SE	46.6 ± 3.0	47.4 ± 6.2	0.8 ± 5.2	49.9 ± 1.9	68.9 ± 1.6	19.0 ± 2.3***	18.2 ± 4.9	0.0078
Median (25–75%)	49.2 (43.7–51.2)	50.9 (35.2–554.5)	−0.05 (−11.2–2.1)	46.7 (43.7–55.9)	67.6 (66.5–74.0)	22.1 (13.1–27.9)		
**Protein % of total kcal**								
Mean ± SE	19.5 ± 2.8	18.9 ± 2.1	−0.7 ± 1.8	14.8 ± 0.6	14.5 ± 0.4	−0.3 ± 0.7	−0.4 ± 2.0	0.7333
Median (25–75%)	16.5 (14.5–26.9)	18.4 (15.4–21.9)	0.7 (−3.4–3.2)	14.4 (13.5–16.5)	14.6 (12.9–15.7)	0.5 (−1.6–1.0)		
**Dietary cholesterol (mg/day)**								
Mean ± SE	217.1 ± 44.1	188.2 ± 37.8	−28.9 ± 55.6	214.2 ± 24.0	7.5 ± 4.2	−206.7 ± 24.3***	−177.8 ± 52.1	0.0343
Median (25–75%)	186.3 (109.8–317.9)	188.5 (91.6–222.2)	−42.9 (−173.6–104.1)	186.7 (132.4–269.2)	0.0 (0.0–1.7)	−164.4 (−254.0– −123.3)		
**Dietary Fiber per 1,000 kcal**								
Mean ± SE	16.6 ± 1.9	14.7 ± 1.4	−1.9 ± 1.6	12.5 ± 1.3	30.7 ± 1.4	18.1 ± 1.7***	20.0 ± 2.6	0.0108
Median (25–75%)	15.2 (12.1–20.2)	14.5 (9.7–18.0)	−0.4 (−4.5–2.1)	23.0 (8.3–14.3)	29.2 (27.4–35.6)	19.1 (11.4–23.4)		

^*^p < 0.05 for within-group change. ^***^p < 0.0001 for within-group change.

All isoflavones increased significantly (*p* < 0.0001) within the WFPB intervention group (Table 2). The WFPB intervention participants increased their daily consumption of total isoflavones from a mean of 0.8 ± mg/day to 14.5 ± 3.2 mg/day (*p* < 0.0001). Within the WFPB group, daidzein, genistein, glycitein, and total isoflavones, increased significantly on average by 5.3 ± 1.2 mg/day, 7.3 ± 1.7 mg/day, 1.2 ± 0.3 mg/day, and 13.7 ± 3.2 mg/day, respectively (all *p* < 0.0001). No significant differences were observed within the control group. Between group differences in change were significant for each isoflavone and the total isoflavones (all *p* < 0.01).

**Table 2:** Isoflavones.

	Control (CON) *n* = 10			Intervention (WFPB) *n* = 19			Between group differences in change at week 8	
	Baseline	8 weeks	Difference	Baseline	8 weeks	Difference	Differences	*p*-value
**Daidzein (mg)**								
Mean ± SE	1.5 ± 0.7	0.2 ± 0.1	−1.2 ± 0.7	0.3 ± 0.1	5.6 ± 1.2	5.3 ± 1.2***	−6.5 ± 1.4	0.0001
Median (25–75%)	0.3 (0.1–1.7)	0.2 (0.1–0.2)	−0.07 (−1.6–0.05)	0.1 (0.1–0.3)	3.9 (0.9–8.7)	3.9 (0.8–8.5)		
**Genistein (mg)**								
Mean ± SE	2.2 ± 1.2	0.3 ± 0.1	−1.9 ± 1.2	0.4 ± 0.2	7.6 ± 1.7	7.3 ± 1.7***	−9.2 ± 2.1	0.0008
Median (25–75%)	0.2 (0.1–2.6)	0.2 (0.1–0.3)	0.0 (−2.4–0.1)	0.1 (0.1–0.3)	5.7 (1.0–14.3)	5.2 (0.9–14.1)		
**Glycitein (mg)**								
Mean ± SE	0.3 ± 0.1	0.0 ± 0.0	−0.2 ± 0.1	0.1 ± 0.0	1.2 ± 0.3	1.2 ± 0.3***	−1.4 ± 0.3	0.0008
Median (25–75%)	0.0 (0.0–0.4)	0.0	0.00 (−0.4–0.0)	0.0	0.8 (0.1–1.9)	0.7 (0.1–1.8)		
**Total isoflavones (mg)**								
Mean ± SE	3.9 ± 2.0	0.6 ± 0.2	−3.3 ± 2.1	0.8 ± 0.4	14.5 ± 3.2	13.7 ± 3.2***	−17.1 ± 3.8	0.0012
Median (25–75%)	0.4 (0.1–4.7)	0.3 (0.2–0.5)	−0.1 (−4.3–0.1)	0.2 (0.1–0.7)	10.8 (1.9–24.7)	10.0 (1.9–24.4)		

^***^p < 0.0001 for within-group change.

Table 3 summarizes the observed amounts of omega PUFAs, including linolenic acid (n-3 PUFA), total n-3 PUFAs, linoleic acid (n-6 PUFA), and linoleic acid: total n-3 PUFAs ratio (n-6:n-3). Within the WFPB group, the consumption of all PUFAs changed significantly (*p* < 0.001). The WFPB intervention significantly decreased the linoleic acid to total omega-3 PUFAs ratio (n-6:n-3) from a mean of 9.3 ± 0.9 to 3.7 ± 0.3 (*p* < 0.0001). Within the WFPB group, linoleic acid (n-6) consumption decreased by 3.8 ± 1.3 g/day (*p* = 0.0095), from 12.8 ± 1.1 g/day to 9.0 ± 0.6 g/day; the total n-3 consumption increased by 1.1 ± 0.3 g/day (*p* = 0.0005), from 1.6 ± 0.2 g/day to 2.7 ± 0.3 g/day as a result of increased non-marine alpha-linolenic acid consumption. Within the control group, the only significant change in omega PUFAs was decreased intake of linolenic acid from 2.4 ± 0.5 g/day to 1.6 ± 0.4 g/day (*p* = 0.0488). Between group differences in change were significant for linolenic acid (*p* = 0.0009), total n-3 PUFA (*p* = 0.0031), and the n-6:n-3 ratio (*p* = 0.0015), but not linoleic acid.

**Table 3:** Omega-3 and linoleic (omega-6) fatty acids.

	Control (CON) *n* = 10			Intervention (WFPB) *n* = 19			Between group differences in change at week 8	
	Baseline	8 weeks	Differences	Baseline	8 weeks	Differences	Differences	*p*-value
**Linolenic acid (g/day)**								
Mean ± SE	2.4 ± 0.5	1.6 ± 0.4	−0.8 ± 0.3*	1.4 ± 0.2	2.7 ± 0.3	1.2 ± 0.3**	−2.0 ± 0.4	0.0009
Median (25–75%)	1.9 (1.7–3.1)	1.4 (1.2–1.8)	−0.7 (−1.2– −0.4)	1.4 (0.9–1.9)	2.3 (1.9–3.4)	1.2 (0.5–2.1)		
**Total omega-3 FA (g/day)**								
Mean ± SE	2.6 ± 0.5	1.9 ± 0.5	−0.7 ± 0.4	1.6 ± 0.2	2.7 ± 0.3	1.1 ± 0.3**	−1.7 ± 0.5	0.0031
Median (25–75%)	2.0 (1.8–3.0)	1.4 (1.3–1.8)	−0.7 (−1.4–0.1)	1.4 (0.9–2.0)	2.3 (1.9–3.4)	1.0 (0.3–1.5)		
**Linoleic acid (g/day)**								
Mean ± SD	15.5 ± 3.3	11.6 ± 1.8	−3.9 ± 2.9	12.8 ± 1.1	9.0 ± 0.6	−3.8 ± 1.3**	−0.1 ± 2.7	0.5556
Median (25–75%)	15.1 (8.2–18.0)	12.5 (7.1–16.4)	−4.9 (−6.4–1.9)	11.3 (9.1–16.8)	8.7 (7.1–10.9)	−3.2 (−7.8–0.5)		
**Linoleic acid/total omega-3 FA ratio**								
Mean ± SD	7.1 ± 1.1	7.6 ± 1.2	0.5 ± 1.3	9.3 ± 0.9	3.7 ± 0.3	−5.5 ± 0.8**	6.0 ± 1.5	0.0015
Median (25–75%)	8.4 (3.5–9.3)	9.1 (3.5–9.2)	0.7 (0.2–3.5)	9.2 (7.6–10.3)	3.3 (2.3–5.0)	−5.4 (−6.8– −2.8)		

^*^p < 0.05 for within-group change. ^**^p < 0.01 for within-group change.

## 4 Discussion

To the best of our knowledge, this is the first description of isoflavone, linoleic acid (n-6 PUFA), and omega-3 PUFA intake in a WFPB diet among women with breast cancer. We observed that our WFPB intervention increased the consumption of isoflavones and non-marine alpha-linolenic acid (n-3 PUFA) while decreasing linoleic acid (n-6 PUFA). The linoleic acid to omega-3 PUFAs (n-6:n-3) ratio decreased from 9.3:1 to 3.7:1.

The baseline isoflavone intake in our cohort of women with metastatic breast cancer was very low but is consistent with other studies, which have found that Americans generally consume less than 1 mg/day (28, 29). In China and Japan, by contrast, some estimates of mean daily isoflavone intake have ranged from 20-50 mg/day or more (30, 31). Trials of isoflavone supplements on various outcomes utilize supplements of 25–300 + mg/day (32, 33). Thus, the total isoflavones consumed in our study, 14.5 mg/day, on a whole-food, plant-based diet with the inclusion of modest amounts of soy foods (~1 meal with a soy component every other day) could be described as no more than a moderate amount in a global context, even though it was roughly 20-fold more than the intervention group consumed at baseline.

Among observational studies, the beneficial inverse association of isoflavone intake and breast cancer diagnosis, recurrence, and mortality appears only after intake exceeds 10 mg/day (16, 34). While there remain many uncertainties regarding the effect of isoflavones on breast cancer, these observational studies offer support for the idea that increasing isoflavone to the level observed in this intervention is likely to be safe and potentially may offer benefits. Because our study was limited to 8 weeks and included many more changes than just isoflavones, we cannot draw any conclusions from our study regarding the increased isoflavones and their potential effect on cancer progression.

Apart from their effect on cancer progression, a meta-analysis of 16 pooled randomized control trials showed that isoflavone intake improved overall cognitive function and memory in adults via mechanisms involving decreased inflammation and oxidative stress (35). This is particularly relevant given that cognitive impairment occurs in 45% of breast cancer patients receiving chemotherapy (36). While increasing isoflavone intake, intervention group participants in this study reported clinically and statistically significant increases in perceived cognitive function, including in areas of memory, concentration, and attention, as measured across several validated questionnaires (27). It is not possible to attribute this strictly to isoflavones, however, given the plethora of related dietary changes and metabolic changes occurring simultaneously.

Concurrent with the changes in isoflavones, the WFPB diet had a significantly lower n-6:n-3 PUFAs ratio resulting from higher n-3 PUFAs and lower linoleic acid (n-6 PUFA) consumption. The effect of these changes on breast cancer progression is inconsistent across studies, but compelling animal research (37) along with some observational studies (38–41) suggest that if there is an effect, it is likely to lower breast cancer risks. However, the issue is further complicated by the potentially different effects of different sources of n-3 PUFAs. Here, study participants increased plant-sourced alpha-linolenic acid (ALA) intake without increasing intake of docosahexaenoic acid (DHA) or eicosapentaenoic acid (EPA), the downstream metabolites of ALA that are commonly consumed preformed in marine animal foods (fish and seafood). While research has often focused on DHA and EPA intake instead of ALA, a case–control study found that women with higher levels of ALA in breast tissue were less likely to have breast cancer (38). Further, the significantly decreased omega-6 intake is likely to enhance the conversion of ALA to EPA and DHA, increasing their serum levels (42–44).

There has also been evidence relating n-6 and n-3 PUFAs intake to cardiometabolic outcomes (22, 45), though there are inconsistent findings here as well, particularly because of observations that higher n-6 PUFA intake is associated with lower cardiovascular risks (46). Our dietary intervention, lower in n-6 PUFA and higher in n-3 PUFA, resulted in multiple cardiometabolic and hormonal improvements, including intentional weight loss without signs of disease progression, reduced total and LDL cholesterol, insulin resistance, free testosterone, and IGF-1 (26). These cannot be attributed solely to changes in fatty acid intake, however, due to the multitude of simultaneous changes in nutrient intakes.

Our study has numerous strengths and limitations. We successfully implemented much larger dietary changes than many other nutrition interventions, in part because of the provided meals. This may however limit generalizability. Patients adopting a WFPB diet on their own may not make such large, rapid changes. Another limitation to generalizability is that different plant-based diets can be constructed differently, with differing amounts of soy foods, chia and flax seeds, etc. But while there will be variation in intake, this descriptive analysis of our study intervention provides a framework for the types of nutrient changes that might be expected with a WFPB diet, which has not previously been described.

In conclusion, the findings from the current study show that transitioning to a WFPB diet inclusive of small amounts of soy foods resulted in significantly increased intake of isoflavones, n-3 PUFAs, and reduced linoleic acid (n-6 PUFA) intake and n-6:n-3 PUFA ratio. While these changes were observed along with numerous cardiometabolic and quality-of-life benefits in this trial, the implications for breast cancer progression and survival require further study.

## Data availability statement

The raw data supporting the conclusions of this article will be made available by the authors, without undue reservation.

## Ethics statement

The studies involving humans were approved by University of Rochester Research Subjects Review Board. The studies were conducted in accordance with the local legislation and institutional requirements. The participants provided their written informed consent to participate in this study.

## Author contributions

JL: Writing – original draft, Data curation, Visualization. EKC: Conceptualization, Data curation, Investigation, Methodology, Project administration, Resources, Supervision, Writing – review & editing, Formal analysis. EvC: Data curation, Formal analysis, Methodology, Resources, Writing – review & editing. LB: Project administration, Writing – review & editing, Data curation, Formal analysis, Investigation. NW: Investigation, Methodology, Project administration, Resources, Writing – review & editing, Data curation. LP: Conceptualization, Methodology, Project administration, Resources, Supervision, Visualization, Writing – review & editing. TC: Conceptualization, Funding acquisition, Investigation, Methodology, Project administration, Resources, Supervision, Writing – review & editing.
