# Supplementary material for: Changes in the consumption of isoflavones, omega-6, and omega-3 fatty acids in women with metastatic breast cancer adopting a whole-food, plant-based diet: post-hoc analysis of nutrient intake data from an 8-week randomized controlled trial
Source: Front Nutr. 2024 Mar 21;11:1338392. doi: 10.3389/fnut.2024.1338392 (PMC10991800; doi:10.3389/fnut.2024.1338392)
Supplement: Supplementary file 1 [file Table_1.DOCX]

***Supplementary Material***

# Menu

**Week 1**

Day 1

Breakfast Muesli with Berries

Lunch Ocean Chickpea Sandwich with Sandwich Fixings and Sweet Potato Fries

Dinner Mushroom Stew with Roasted Green Beans and onions

Side Southern Kale

Day 2

Breakfast Steel Cut Oats with a Fruit Cup

Lunch Mushroom Stew with Roasted Green Beans and Onions

Dinner Eggplant “Parmesan” with Whole Grain Pasta and Marinara Sauce

Side Salad with Dressing

Day 3

Breakfast Vanilla Chia Oats with Berries

Lunch Eggplant “Parmesan” with Whole Grain Pasta and Marinara Sauce

Dinner Ocean Chickpea Sandwich with Sandwich Fixings and Sweet Potato Fries

Side Grilled Veggies

Day 4

Breakfast Banana Flax Muffin with Fruit

Lunch Vegetable Barley Soup with Side

Dinner Soba Peanut Noodles with Edamame, Broccoli and Cucumber

Side Roasted Broccoli and Cauliflower

Day 5

Breakfast Muesli with Berries

Lunch Soba Peanut Noodles with Edamame, Broccoli and Cucumber

Dinner “Shepard’s Pie” with Parsnip Crust and Asparagus

Side Salad with Dressing

Day 6

Breakfast Steel Cut Oats with Fruit Cup

Lunch Tempeh Goulash with Steamed Broccoli

Dinner Vegetable Barley Soup with Side

Side Veggie Pasta Salad

Day 7

Breakfast Vanilla Chia Oats with Berries

Lunch Tempeh Goulash with Broccoli

Dinner “Shepard’s Pie” with Parsnip Crust and Asparagus

Side Roasted Zucchini, Peppers, and Onions

**Week 2**

Day 1

Breakfast Banana Flax Muffin with Fruit

Lunch Sweet Potato Enchiladas with Side

Dinner Mediterranean White Bean Soup with Side

Side Salad with Dressing

Day 2

Breakfast Muesli with Berries

Lunch Veggie Burger with Sandwich Fixings and Roasted Red Potatoes

Dinner Sweet Potato Enchiladas with Brown Rice

Side Greens and Beans

Day 3

Breakfast Steel Cut Oats with Fruit Cup

Lunch Mediterranean White Bean Soup with a Side

Dinner Veggie Burger with Sandwich Fixings and Roasted Red Potatoes

Side Roasted Broccoli

Day 4

Breakfast Vanilla Chia Oats with Berries

Lunch Curried Vegetable Stew with Farro

Dinner Moussaka with Pinenut Cream with Side

Side Rainbow Salad

Day 5

Breakfast Banana Flax Muffin with Fruit Cup

Lunch Pineapple Stirfry with Broccoli and Brown Rice

Dinner Chickpea Loaf with Roasted Root Vegetables and Green Veggie

Side Roasted Asparagus and Carrots

Day 6

Breakfast Muesli with Berries

Lunch Curried Vegetable Stew with Farro

Dinner Pineapple Stirfry with Broccoli and Brown Rice

Side Sautéed Kale

Day 7

Breakfast Steel Cut Oats with Fruit Cup

Lunch Chickpea Loaf with Roasted Root Vegetables and Green Veggie

Dinner Moussaka with Pinenut Cream with Side

Side Salad with Dressing

**Week 3**

Day 1

Breakfast Muesli with Berries

Lunch Veggie Burger with Sandwich Fixings and Roasted Red Potatoes

Dinner Sweet Potato Enchiladas

Side Swiss Chard

Day 2

Breakfast Steel Cut Oats with Walnuts and Flax and Fruit Cup

Lunch Lasagna

Dinner Veggie Burger with Sandwich Fixings and Roasted Red Potatoes

Side Salad with Dressing

Day 3

Breakfast Vanilla Chia Oats Parfait with Berries

Lunch Sweet Potato Enchiladas

Dinner Lasagna

Side Grilled Veggies

Day 4

Breakfast Banana Flax Muffin with Fruit Cup

Lunch Mushroom Bok Choy Soup

Dinner Mexican Rice

Side Roasted Broccoli Red Peppers and Almonds

Day 5

Breakfast Muesli with Berries

Lunch “Mac N Cheese” with Broccoli

Dinner Moroccan Chickpea Stew with Farro

Side Salad with Dressing

Day 6

Breakfast Steel Cut Oats with Walnuts and Flax and Fruit Cup

Lunch Mexican Rice

Dinner Mushroom Bok Choy Soup

Side Roasted Brussel Sprouts with Applesauce

Day 7

Breakfast Vanilla Chia Oats Parfait with Berries

Lunch Moroccan Chickpea Stew with Farro

Dinner “Mac N Cheese” with Broccoli

Side Green Beans and Mushrooms

**Week 4**

Day 1

Breakfast Banana Flax Muffin with Fruit Cup

Lunch Stir-Fried Eggplant, Peppers and Tofu

Dinner “Sloppy Joes” with Roll and Roasted Red Potatoes

Side Salad with Dressing

Day 2

Breakfast Muesli with Berries

Lunch “Sloppy Joes” with Roll and Roasted Red Potatoes

Dinner Split Pea with Grilled Zucchini and Yellow Squash

Side Grilled Veggies

Day 3

Breakfast Steel Cut Oats with Walnuts and Flax and Fruit Cup

Lunch Split Pea with Grilled Zucchini and Yellow Squash

Dinner Stir-Fried Eggplant, Peppers and Tofu

Side Roasted Squash

Day 4

Breakfast Vanilla Chia Oats Parfait with Berries

Lunch Spring Stew

Dinner Ocean Chickpea with Sweet Potato Fries

Side Rainbow Salad

Day 5

Breakfast Banana Flax Muffin with A Fruit Cup

Lunch “Fried” Vegetable Rice

Dinner Eggplant “Meatballs” with Marinara Sauce and Whole Wheat Pasta

Side Roasted Asparagus and Carrots

Day 6

Breakfast Muesli with Berries

Lunch Spring Stew

Dinner Eggplant “Meatballs” with Marinara Sauce and Whole Wheat Pasta

Side Sautéed Kale

Day 7

Breakfast Steel-Cut Oats with Walnuts and Flax with Fruit Cup

Lunch Ocean Chickpea with Sweet Potato Fries

Dinner “Fried” Vegetable Rice

Side Salad with Dressing
